# Application of trio-based whole-exome sequencing in fetal ultrasound anomalies: a single-center retrospective study of 454 cases

**DOI:** 10.3389/fgene.2025.1662801

**Published:** 2025-11-24

**Authors:** Dongyi Yu, Dairong Feng, Jiangbo Qu, Lei Nie, Qian Liu, Lu Gao, Wenzhen An, Na Liu, Yuying Fang

**Affiliations:** 1 Center for Medical Genetics and Prenatal Diagnosis, Shandong Provincial Maternal and Child Health Care Hospital Affiliated to Qingdao University, Jinan, Shandong, China; 2 Key Laboratory of Birth Defect Prevention and Genetic Medicine of Shandong Health Commission, Jinan, Shandong, China; 3 Key Laboratory of Maternal & Fetal Medicine of the National Health Commission, Jinan, Shandong, China; 4 Medical Management Service Center of Health Commission of Shandong Province, Jinan, Shandong, China; 5 Medical Department, Zhejiang Biosan Biochemical Technologies Co., Ltd, Hangzhou, Zhejiang, China

**Keywords:** fetal ultrasound anomalies, prenatal diagnosis, whole-exome sequencing, microarray analysis, chromosome disorders, single gene disorders

## Abstract

This study assessed the diagnostic effectiveness of trio-WES compared to CMA in fetuses with ultrasound anomalies and explored optimal prenatal testing strategies. A retrospective review included 454 fetuses who underwent trio-WES and/or CMA between 2020 and 2023. Cases were divided into four groups and 19 subgroups based on improvements in diagnosing ultrasound anomalies, demonstrating that trio-WES offers greater diagnostic value than CMA—especially for skeletal and multisystem defects, as well as ultrasound findings used to determine diagnostic yields. Trio-WES achieved a diagnostic yield of 22.7% (103/454), exceeding CMA by 17%. The highest diagnostic rates were observed in skeletal anomalies (39.2%) and multisystem anomalies (29.1%). Subgroup analysis showed higher yields in crystalline lens anomalies (60.0%) and cardiac rhabdomyoma (57.1%). Trio-WES significantly enhances prenatal diagnosis of ultrasound anomalies and provides additional diagnostic insights over CMA, particularly for skeletal, multisystem defects, and specific structural subgroups. Importantly, trio-WES helps clarify the mechanisms of ROH and assess its pathogenicity, aiding in detecting imprinted disorders. These findings support including trio-WES in prenatal testing protocols for congenital malformations and establish a framework for its clinical application.

## Introduction

Congenital disorders are a significant cause of perinatal morbidity and mortality. The World Health Organization’s 2023 report indicated that approximately 6% of newborns worldwide are affected by congenital diseases, leading to around 240,000 deaths during the first 28 days of life each year (WHO, 2023). These disorders range from minor anomalies to severe, life-threatening conditions. The genetic basis of many of these conditions is complex and remains largely unexplored. While prenatal ultrasound is capable of identifying specific abnormalities (Campbell et al., 2005), determining their associated genetic origins presents significant challenges. The diagnostic methods for prenatal diagnosis, including karyotyping and CMA, are typically used for confirmation. While karyotyping effectively detects numerical and structural chromosomal abnormalities, it is limited by long processing times and its inability to resolve CNVs smaller than 5 Mb. CMA and low-coverage genome sequencing techniques, such as CNV-seq, offer enhanced CNV detection capabilities, improving sensitivity by approximately 6% (Wapner et al., 2012). Despite these advancements, more than 60% of pregnancies with structural abnormalities still lack a definitive genomic diagnosis, complicating genetic counseling and clinical management (Petrovski et al., 2019).

The rapid advancement of next-generation sequencing (NGS) technologies has revolutionized genetic diagnostics for fetal abnormalities detected by prenatal ultrasound (Dixon et al., 2011; Emms et al., 2022; Moresco et al., 2022; Zhou et al., 2023). WES, which targets the protein-coding regions of the genome, offers a more comprehensive approach to identifying single-nucleotide variations (SNVs) as well as insertions and deletions (indels) associated with genetic disorders. This method has proven effective in elucidating the genetic causes of fetal anomalies detected by ultrasound, providing valuable insights into genotype-phenotype correlations (Hopkins et al., 2020; Monaghan et al., 2020). Despite its potential, the clinical application of this sequencing technique in prenatal diagnostics remains underexplored. The ongoing accumulation of phenotype and genotype data is crucial for enhancing the accuracy of genetic interpretations, advancing our understanding of the molecular mechanisms underlying fetal structural anomalies, and enabling more precise genetic counseling.

In this study, we retrospectively analyzed the clinical and trio-WES results of 454 fetuses with ultrasound abnormalities. We investigated the molecular diagnostic yield of prenatal trio-WES for various fetal ultrasound anomalies by incorporating a broad range of clinical phenotypes. We identified pathogenic genes and variations associated with these conditions. Additionally, we conducted a comparative analysis of the diagnostic performance of trio-WES and CMA across different types of ultrasound abnormalities, providing further insight into the utility of trio-WES in prenatal genetic diagnostics.

## Materials and methods

### Study design and participants

This was a retrospective study of families who experienced fetal abnormalities or fetal loss and were referred to the Center of Medical Genetics and Prenatal Diagnosis at Shandong Provincial Maternal and Child Health Hospital, affiliated to Qingdao University, from January 2020 to February 2023. The inclusion criteria included singleton pregnancies with available fetal samples (amniotic fluid or chorionic villi) and parental consent for trio-WES analysis. Cases were excluded if they had incomplete clinical or genetic data, lacked parental samples, or if the family chose not to undergo genetic testing. Fetal anomalies were classified into five groups based on ultrasound findings: (1) structural anomalies, (2) fetal growth restriction (FGR), (3) stillbirth (defined as fetal death at or after 20 weeks of gestation without obvious ultrasound structural anomalies), and (4) ultrasound soft markers (USMs).

Furthermore, the group of structural anomalies was further divided into 15 subgroups, including anomalies of the multisystem, skeletal system, neurological system, genitourinary system, cardiovascular system, increased nuchal translucency or cystic hygroma (IncrNT/CH), craniofacial system, digestive system, amniotic fluid volume-oligohydramnios/polyhydramnios (AFV-O/P), abdomen, situs inversus, hydrops, respiratory system, cardiac rhabdomyoma, and crystalline lens anomalies. It is important to note that cardiac rhabdomyoma and crystalline lens anomalies suggest a high prior probability of a genetic diagnosis. These cases were analyzed separately from the subgroups within the cardiovascular system or craniofacial anomalies to prevent their inherently high diagnostic yield from skewing the results for the related subgroups and to allow a distinct evaluation of trio-WES performance in these unique, high-yield scenarios.

### Trio-based whole-exome sequencing and data analysis

Trio-WES was conducted on DNA extracted from fetal samples and parental blood samples. Following standard protocols, DNA was isolated using the column-based TIANGEN DP316 Micro Sample Genome DNA Extraction Kit. DNA quality and concentration were evaluated using the Nanodrop One microspectrophotometer. Samples were hybridized with Roche KAPA HyperExome v2 probes for whole-exome capture, targeting all annotated coding exons of genes related to fetal abnormalities, along with their adjacent ±10 bp non-coding regions. High-throughput sequencing was then performed on the MGI DNBSEQ-T7 platform. The sequencing achieved a coverage of ≥99% of the target regions, with a depth of ≥20× at over 99% of positions. Sequencing data were aligned to the human reference genome GRCh37/hg19 using BWA, and variant calling for SNVs and small indels was carried out using the GATK best practices pipeline.

Variant sites were annotated and filtered using ANNOVAR. Candidate variants were filtered against population databases, including the 1000 Genomes Project (https://www.internationalgenome.org) and gnomAD (https://gnomad.broadinstitute.org). Bioinformatics tools, such as SIFT (https://sift.bii.a-star.edu.sg/), PolyPhen-2 (http://genetics.bwh.harvard.edu/pph2/), MutationTaster (https://www.mutationtaster.org/), the Combined Annotation Dependent Depletion (CADD) score (https://cadd.gs.washington.edu/), and SpliceAI Lookup (https://spliceailookup.broadinstitute.org/) were utilized to predict and analyze the pathogenicity of candidate variants.

For detecting copy number variants (CNVs) from WES data, the DNAcopy R package was used to implement the circular binary segmentation (CBS) algorithm, which segments copy number data to identify genomic regions with abnormal copy number at a resolution of 100 kb. All clinically reported CNVs were orthogonally validated through quantitative PCR (qPCR). A subset of these WES-based CNV calls, especially those of clinical importance and smaller size, were validated using qPCR; however, systematic orthogonal validation was not performed for all calls, which is a limitation of the study.

### Chromosomal microarray analysis

Chromosomal microarray analysis (CMA) was conducted using Affymetrix CytoScan 750K arrays (Applied Biosystems, Thermo Fisher Scientific), which include over 750,000 markers—about 200,000 SNP markers for genotype information and 550,000 non-polymorphic probes for copy number detection—distributed across the entire genome. The array has a probe density of approximately one marker per 4.1 kb, allowing for detection of copy number variants and copy-neutral loss of heterozygosity (CN-LOH). Data analysis was carried out using the Chromosome Analysis Suite (ChAS) software (v4.3) with recommended settings for prenatal samples.

### Variant interpretation and classification

Variants identified through WES were filtered for high-quality calls based on criteria such as read depth, allele frequency, and quality scores. The pathogenicity of variants was assessed according to the American College of Medical Genetics and Genomics (ACMG) guidelines and classified into the following categories: pathogenic (P), likely pathogenic (LP), variant of uncertain significance (VUS), likely benign (LB), and benign (B).

Clinical reports provided to families included variants associated with the clinical phenotype, consistent with the inheritance pattern, and supported by sufficient evidence of pathogenicity. In certain cases, variants of uncertain significance (VUS) were reported, particularly in autosomal recessive conditions where the VUS was found *in trans* with a pathogenic or likely pathogenic variant and was associated with abnormal fetal phenotypes.

Variants not directly related to the primary indication for fetal testing, but potentially linked to severe childhood-onset conditions, were identified as incidental findings. These findings were discussed with patients during the pre-test informed consent process, allowing them to decide whether to receive reports on these variants.

### Data statistics

Statistical analysis was carried out using SPSS version 27.0. Pearson’s chi-square test was used to compare the diagnostic performance of WES versus CMA across various phenotypic subgroups. A *p*-value of less than 0.05 was considered statistically significant.

## Results

### Participants characteristics

**FIGURE 1:**
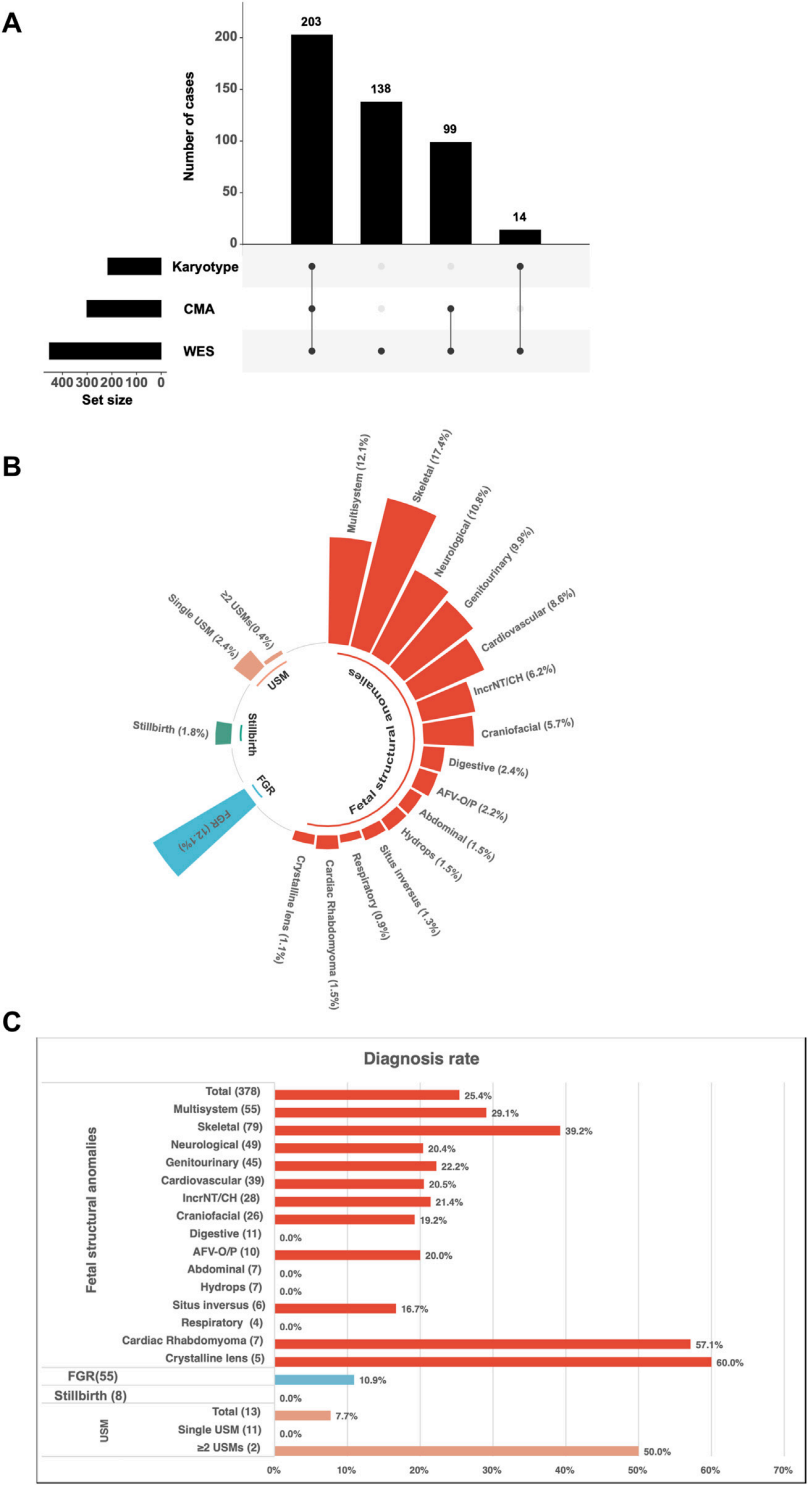
Study enrollment and diagnostic characteristics of fetuses with ultrasound anomalies undergoing trio whole-exome sequencing (trio-WES). **(A)** Study recruitment and testing strategy. A total of 454 fetuses were enrolled, including 203 who underwent karyotyping, CMA, and trio-WES, 138 who underwent trio-WES alone, and 113 (99 + 14) who underwent trio-WES combined with chromosomal microarray analysis (CMA) or karyotyping. **(B)** Distribution of ultrasound anomaly types among the enrolled fetuses. **(C)** Diagnostic yield of trio-WES across different categories of ultrasound anomalies. Abbreviations: AFV-O/P, amniotic fluid volume-oligohydramnios/polyhydramnios; IncrNT, increased nuchal translucency; CH, cystic hygroma; USMs, ultrasound soft markers; FGR, fetal growth restriction.

This retrospective cohort comprised 454 families undergoing trio-WES, including 138 families who underwent trio-WES alone, 113 families who underwent trio-WES combined with CMA or karyotyping, and 203 families who underwent karyotyping, CMA, and trio-WES. (Figure 1A). The enrolled families were classified into four primary phenotypic categories based on fetal ultrasound findings: structural anomalies (378/454, 83.3%), FGR (55/454, 12.1%), USMs (13/454, 2.8%), and stillbirth (8/454, 1.8%) (Figures 1B,C). The structural anomalies category encompassed a range of conditions, including multisystem anomalies (55/454, 12.1%), skeletal anomalies (79/454, 17.4%), neurological anomalies (49/454, 10.8%), genitourinary anomalies (45/454, 9.9%), cardiovascular anomalies (39/454, 8.6%), IncrNT/CH (28/454, 6.2%), craniofacial anomalies (26/454, 5.7%), digestive anomalies (11/454, 2.4%), AFV-O/P (10/454, 2.2%), abdominal anomalies (7/454, 1.5%), hydrops (7/454, 1.5%), situs inversus (6/454, 1.3%), respiratory anomalies (4/454, 0.9%), cardiac rhabdomyoma (7/454, 1.5%) and crystalline lens abnormalities (5/454, 1.1%) (Figures 1B,C). The USMs category was further divided into single USMs (11/454, 2.4%) and ≥2 USMs (2/454, 0.4%) (Figures 1B,C).

### Diagnostic yields by trio-WES in phenotypic categories

The diagnostic yields of trio-WES varied across different phenotypic categories in a cohort of 454 fetuses with ultrasound anomalies (Figure 1C). Among structural anomalies, skeletal system anomalies exhibited the highest diagnostic yield (31/79, 39.2%), followed by multisystem anomalies (16/55, 29.1%), genitourinary anomalies (10/45, 22.2%), IncrNT/CH (6/28, 21.4%), cardiovascular anomalies (8/39, 20.5%), neurological anomalies (10/49, 20.4%), AFV-O/P abnormalities (2/10, 20.0%), craniofacial anomalies (5/26, 19.2%), and situs inversus (1/6, 16.7%) (Figure 1C).

In other ultrasound anomaly groups, the diagnostic yields of trio-WES were as follows: 10.9% (6/55) for FGR, 60% (3/5) for crystalline lens anomalies, 57.1% (4/7) for cardiac rhabdomyoma, and 50% (1/2) for multiple USMs. No clinically relevant variants were identified in the stillbirth category (0/8) (Figure 1C).

### Comparison of diagnostic yields between Trio-WES and CMA across phenotypic categories

**FIGURE 2:**
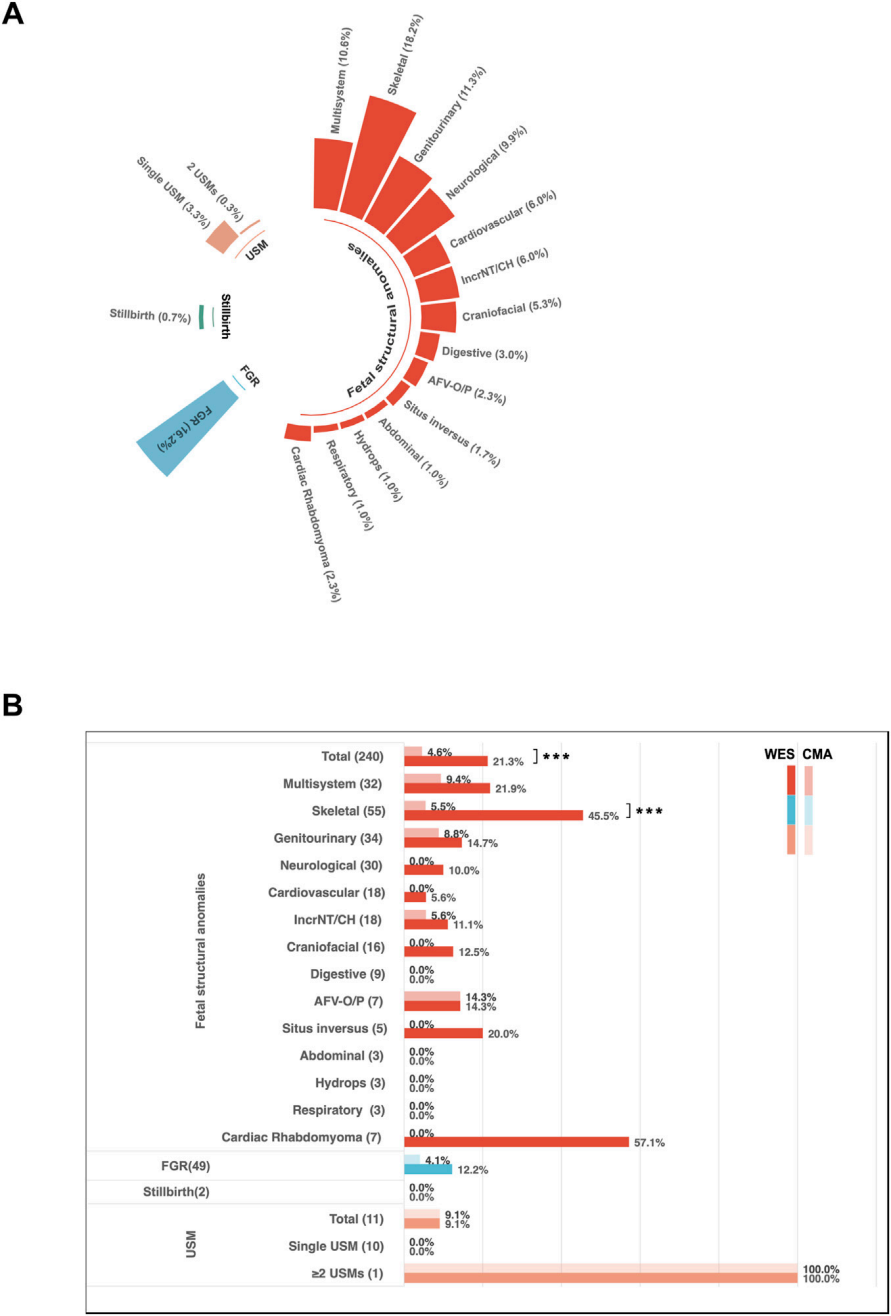
Diagnostic characteristics of fetuses with ultrasound anomalies undergoing trio-WES and chromosomal microarray analysis (CMA). **(A)** Distribution of ultrasound anomaly types among 302 (203 + 99) fetuses who underwent both CMA and trio-WES. **(B)** Diagnostic yields for different categories of ultrasound anomalies among the 302 cases. Dark shading indicates the diagnostic yield from WES, while light shading represents the diagnostic yield from CMA. Abbreviations: AFV-O/P, Amniotic Fluid Volume-Oligohydramnios/Polyhydramnios; IncrNT, Increased Nuchal Translucency; CH, Cystic Hygroma; USM, Ultrasound Soft Markers; FGR, Fetal Growth Restriction.

A total of 302 fetuses underwent both CMA and trio-WES, enabling a direct comparison of diagnostic performance. It is important to note that this subgroup represents a cohort selected for dual-platform testing, which may be influenced by clinical factors such as phenotypic severity. Consequently, the diagnostic yields within this subset are not directly equivalent to those from the broader, unselected WES cohort. The subgroup of crystalline lens anomalies was excluded from this comparative analysis because these cases involved trio-WES exclusively without CMA (Figure 2A).

Overall, trio-WES demonstrated higher diagnostic yields compared to CMA across various phenotypic categories. However, statistically significant differences were observed only in the category of structural anomalies (20.2% vs. 4.7%, *p* < 0.001) and in the subgroup of skeletal anomalies (45.5% vs. 5.5%, *p* < 0.001) (Figure 2B). Although not statistically significant, increases in diagnostic yields by trio-WES were also noted in other subgroups, such as multisystem anomalies (21.9% vs. 9.4%), genitourinary system anomalies (14.7% vs. 8.8%), and IncrNT/CH (11.1% vs. 5.5%). Furthermore, diagnostic variants were identified by trio-WES in several phenotypic subgroups where CMA did not yield a positive result. These subgroups included situs inversus (1/5, 20%), craniofacial anomalies (2/16, 12.5%), neurological anomalies (4/40, 10%), cardiovascular anomalies (2/36, 5.6%), and cardiac rhabdomyoma (4/7, 57.1%) (Figure 2B). Given the small sample sizes in these subgroups, these findings are presented as descriptive observations that highlight potential areas where trio-WES may offer unique diagnostic value, warranting further investigation in larger cohorts.

Trio-WES exclusively provided diagnostic insights for several phenotypic subgroups where CMA failed to identify clinically relevant variants, including situs inversus (20%), craniofacial anomalies (12.5%), neurological anomalies (10%), cardiovascular anomalies (5.6%), and cardiac rhabdomyoma (57.1%) (Figure 2B).

### Additional diagnostic yields provided by trio-WES

**FIGURE 3:**
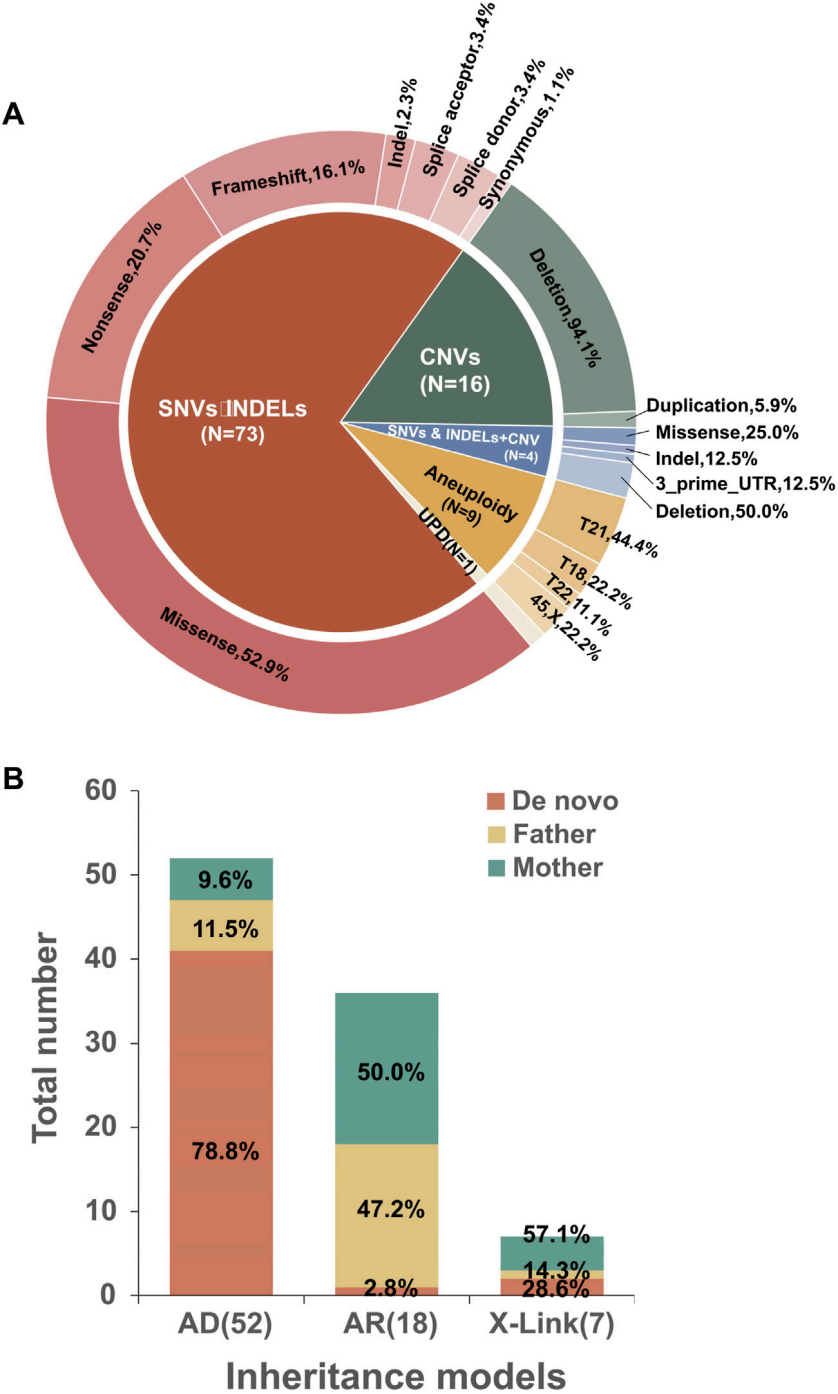
Characterization of molecular variants. **(A)** Distribution of variant types among the 103 cases with positive findings. **(B)** Inheritance models were identified in cases with diagnostic sequence variants, along with the proportion of *de novo* and inherited variants.

Among the 454 enrolled fetuses, 103 had positive genetic findings. Specifically, 73 tested positive for SNVs/indels, four had both SNVs/indels and CNVs, 16 were positive for CNVs alone, nine were identified with aneuploidies, and one exhibited mixed maternal UPD of chromosome 15 (Figure 3A). Twenty-six cases showing aneuploidies, CNVs, or UPD variants identified through trio-WES with or without CMA and/or karyotyping were summarized in Table 1. Seventy-seven cases with SNVs, indels, and CNV variants detected exclusively by trio-WES were summarized in Table 2. In two discordant cases, WES identified an additional 701 kb deletion at 17q21.31 that CMA missed (Case 7). In the other case (Case 24), CMA detected regions of homozygosity (ROH) on chromosome 15, and trio-WES was crucial in determining the parental origin of these alleles, confirming a diagnosis of mixed maternal UPD(15) associated with Prader-Willi syndrome and demonstrating the importance of WES in clarifying the pathogenic mechanism behind CMA findings.

**TABLE 1:** Summary of Aneuploidies and Pathogenic or Likely Pathogenic Copy Number Variants (CNVs) Identified by Trio-based Whole Exome Sequencing (trio-WES) with or without Chromosomal Microarray Analysis (CMA) and/or karyotyping.

Case Id	Ultrasound findings	CMA	WES	Pregnancy outcome
1	Cardiovascular	NA	seq (21) × 3	TOP
2	Cardiovascular	NA	seq (21) × 3; (X) × 3	TOP
3	IncrNT/CH + USM-CPC	NA	seq (18) × 3	TOP
4	IncrNT/CH + USM-EIF	arr (21) × 3	seq (21) × 3	TOP
5	Neurological + Skeletal + Cardiovascular + Genitourinary	NA	seq (22) × 3	TOP
6	Cardiovascular + USM-LVB	NA	seq [hg19]1q44 (244,810,410–249,250,621)×1	TOP
7	Craniofacial	Not detected	seq [GRCh37]del (17) (q21.31q21.31) chr17:g.43893807_44594822del	Lost to follow-up
8	Craniofacial + USM-CPC	NA	seq (18) × 3	TOP
9	FGR	arr [GRCh37]7q11.23x1	seq [GRCh37]7q11.23 (72,717,385–74160325)x1	TOP
10	FGR + Neurological + AFV-P	NA	seq [GRCh37]del (17) (p13.3p13.2)chr17:g.5980_4210447del	TOP
11	Genitourinary	arr [GRCh37]22q11.21 (18,648,856_21,800,471)x1	seq [GRCh37]del (22) (q11.21q11.21) chr22:g.18893817_21562621del	TOP
12	Genitourinary	arr [GRCh37]17q12 (34,822,493_36,243,365)x1	seq [GRCh37]del (17) (q12q12) chr17:g.34806159_36104986del	TOP
13	Genitourinary	arr [GRCh37]17q12 (34822466_36404555)x1	seq [GRCh37]del (17) (q12q12) chr17:g.34842505_36104986del	Lost to follow-up
14	Genitourinary	NA	seq [GRCh37]del (17) (q12q12) chr17:g.34842505_36104986del	Lost to follow-up
15	Genitourinary + AFV-P	arr [GRCh37]17q12 (34822466_36307773)x1	seq [GRCh37]17q12 (34,842,526–36,104,965)x1	Live birth without any abnormality
16	IncrNT/CH	NA	seq(X) × 1	TOP
17	IncrNT/CH	NA	seq [GRCh37]17p11.2 (17,696,253–18,668,175)x1	TOP
18	Neurological	NA	seq (21) × 3	TOP
19	Neurological + Cardiovascular + USM-SUA	arr(X) × 1	seq(X) × 1	Lost to follow-up
20	Neurological + Ocular + USM-HNB	arr [GRCh37] 11p14.3p11.12 (2391919_5589224)x1	seq [GRch37]del (11) (p14.3p11.12) chr11:24010449_50004136del	TOP
21	Skeletal	arr [GRCH37]Xp22.33 (168552_920124)X1	seq [GRCh37]del(Y) (p11.32p11.32) chrY:g.150837_555441del	TOP
22	Skeletal	arr [GRCh37]16p11.2 (29591327_30176508)x1	seq [GRCh37]del (16) (p11.2p11.2) chr16:g.29516772_30199925del	Lost to follow-up
23	Skeletal	arr [hg19]16p11.2 (29,580,020–30,330,881)X1	seq [GRCh37]16p11.2 (29,539,626–30,223,125) × 1	TOP
24	AFV-P	arr [GRCh37]15q22.2q26.1 (60941324_92564506)x2 hmz, 31.623 Mb LOH arr [GRCh37]15q26.2q26.3 (96686105_102397317)x2 hmz, 5.711 Mb LOH	UPD(15)mat	Lost to follow-up
25	FGR + USM-ARSA	arr [GRCh37]17q11.2q12 (30341330_34477480)x1 arr [GRCh37]17q12q21.31 (36496455_40979941)x3	seq [GRCh37]17q11.2q12 (30351696–34431395)x1 seq [GRCh37]17q12q21.31 (36,453,122–40933342)x3	Lost to follow-up
26	USM2-EIF/UTD	arr [GRCh37]9q22.33 (101843486_102341851)x1	seq [GRCh37]9q22.33 (101,867,473–101992722)x1	Live birth with hydronephrosis

Abbreviations: TOP (Termination of Pregnancy).

**TABLE 2:** Summary of diagnostic sequence variants identified by Trio-based Whole Exome Sequencing (trio-WES).

Case ID	Ultrasound findings	Gene	Variants	Molecular consequence	Zygosity	ACMG classification	ACMG criteria	Origin	OMIM diseases	Inheritance mode	Pregnancy outcome
27	Skeletal	*DYNC2H1*	c5256del (p.A17530fs*13) c.9737C>T (pT3246l)	Frameshift Missense	Het Het	LP VUS	PVS1+PM2_P PM2 P + PM3	Mother Father	Short-rib thoracic dysplasia 3 with or without polydactyly (OMIM 613091)	AR	TOP
28	AFV-P	*HRAS*	c.34G>A (p.G12S)	Missense	Het	P	PS2_VS + PS3+PS4+PM1+PM2+PP3	*De novo*	Costello syndrome (218040)	AD	TOP
29	Cardiac rhabdomyoma	*TSC1*	c.1327G>T (p.G443*)	Nonsense	Het	P	PVS1+PS2_M + PM2_P	*De novo*	Tuberous sclerosis-1 (191100)	AD	TOP
30	Cardiac rhabdomyoma	*TSC1*	c.308G>A (p.W103*)	Nonsense	Mosaic	P	PVS1+PS2_M + PS4_P + PM2_P	*De novo*	Tuberous sclerosis-1 (191100)	AD	Lost to follow-up
31	Cardiac rhabdomyoma	*TSC2*	c.5238_5255delCATCAAGCGGCTCCGCCA (p.His1746_Arg1751del)	Indel	Het	P	PS4+PM4+PM2-PP + PS2-VeryStrong	*De novo*	Tuberous sclerosis-2 (613254)	AD	Lost to follow-up
32	Cardiac rhabdomyoma	*TSC2*	c.4351dup (p.R1451Pfs*73)	Frameshift	Het	P	PVS1+PS2+PM2_P	*De novo*	Tuberous sclerosis-2 (613254)	AD	TOP
33	Cardiovascular	*KMT2D*	c.15844C>T (p.R5282*)	Nonsense	Het	P	PVS1+PS2_M + PS4_P + PM2_P	*De novo*	Kabuki syndrome 1 (147920) BCAHH syndrome (620186)	AD	Lost to follow-up
34	Cardiovascular	*CHD7*	c.7957C>T (p.R2653*)	Nonsense	Het	P	PVS1+PM2_PP + PS2_VeryStrong	Mother	CHARGE syndrome (214800) Hypogonadotropic hypogonadism 5 with or without anosmia (612370)	AD	TOP
35	Cardiovascular	*PTPN11*	c.188A>G (p.Y63C)	Missense	Het	P	PS2_M + PS3+PS4+PP2+PP3	Father	LEOPARD syndrome 1 (151100) Noonan syndrome 1 (163950)	AD	TOP
36	Cardiovascular	*MYH7*	c.475G>A (p.D159N)	Missense	Het	LP	PS2+PS4_P + PM2	*De novo*	Cardiomyopathy, dilated, 1S (613426)	AD	TOP
37	Cardiovascular	*KMT2D*	c.12333_12345delAGGTGGAGGAAGC p. (G4112Mfs*4)	Frameshift	Het	P	PVS1+PS2+PM2	*De novo*	Kabuki syndrome 1 (147920)BCAHH syndrome (620186)	AD	TOP
38	Craniofacial	*FGFR3*	c.749C>G (p.P250R)	Missense	Het	P	PS2_VS + PS4+PM2_P + PP1_S + PP3	*De novo*	Achondroplasia (OMIM 100800) Hypochondroplasia (OMIM 146000)	AD	Lost to follow-up
39	Craniofacial	*COL2A1*	c.2678dup (p.A895Sfs*49)	Frameshift	Het	P	PVS1+PS2_M + PM2_P	*De novo*	?Epiphyseal dysplasia, multiple, with myopia and deafness (132450) ?Vitreoretinopathy with phalangeal epiphyseal dysplasia (619248) Achondrogenesis, type II or hypochondrogenesis (200610) Avascular necrosis of the femoral head (608805) Czech dysplasia (609162) Kniest dysplasia (156550) Legg-Calve-Perthes disease (150600) Osteoarthritis with mild chondrodysplasia (604864) Platyspondylic skeletal dysplasia, Torrance type (151209)	AD	TOP
40	Craniofacial	*EFNB1*	c.266G>A (p.C89Y)	Missense	Het	LP	PM1+PM2+PP3+PP4+PS3-pp	Father	Craniofrontonasal dysplasia (304110)	XLD	TOP
41	Crystalline lens	*OCRL*	c.688C>T (p.Arg230Ter)	Nonsense	Hemi	P	PVS1+PM2-PP + PS4-PP	Mother	Achondroplasia (OMIM 100800) Hypochondroplasia (OMIM 146000)	XLR	TOP
42	Crystalline lens	*OTX2*	c.247C>T (p.Gln83Ter)	Nonsense	Het	LP	PVS1+PM2-PP	Father	Microphthalmia, syndromic 5 (610125) Pituitary hormone deficiency, combined, 6 (613986) Retinal dystrophy, early-onset, with or without pituitary dysfunction (610125)	AD	TOP
43	Crystalline lens	*OCRL*	c.740G>A (p.W147*)	Nonsense	Hemi	P	PVS1+PS2_M + PS4_P + PM2_P	*De novo*	Achondroplasia (OMIM 100800) Hypochondroplasia (OMIM 146000)	XLR	TOP
44	Cutaneous + Genitourinary + Skeletal + Craniofacial	*ABCA12*	c.400_403del (p.A134Hfs*2) c.1568del (p.M523Rfs*10)	Frameshift Frameshift	Het Het	P LP	PVS1+PM2_P + PM3 PVS1+PM2_P	Father Mother	Ichthyosis, congenital, autosomal recessive 4B (harlequin) (242500) Ichthyosis, congenital, autosomal recessive 4A (601277)	AR	TOP
45	FGR	*COL2A1*	c.1060G>C (p.G354R)	Missense	Het	LP	PS2_M + PM1+PM2_P + PP3	*De novo*	?Epiphyseal dysplasia, multiple, with myopia and deafness (132450) ?Vitreoretinopathy with phalangeal epiphyseal dysplasia (619248) Achondrogenesis, type II or hypochondrogenesis (200610) Avascular necrosis of the femoral head (608805) Czech dysplasia (609162) Kniest dysplasia (156550) Legg-Calve-Perthes disease (150600) Osteoarthritis with mild chondrodysplasia (604864) Platyspondylic skeletal dysplasia, Torrance type (151210)	AD	Lost to follow-up
46	FGR	*CENPJ*	c.2750_2755del (p.I917_E918del) c.826_830del (p.K276*)	Indel Nonsense	Het Het	VUS LP	PM2_P + PM3+PM4 PVS1+PM2_P	Mother Father	Microcephaly 6, primary, autosomal recessive (608393)	AR	TOP
47	FGR	*RECQL4*	c.2492_2493del (p.H831Rfs*52) c.1879-1G>A	Frameshift Splice donor	Het Het	P VUS	PVS1+PM2_P + PM3+PP1 PVS1_M + PM2_P + PM3	Mother Father	Rothmund-Thomson syndrome, type 2 (268400)	AR	TOP
48	Genitourinary	*PKHD1*	c.6840G>A (p.W2280*) c.6900C>T (p.N2300 =)	Nonsense Synonymous	Het Het	P LP	PM2_P + PM3_P + PVS1 PS3+PM2_P + PM3	*De novo* Mother	Polycystic kidney disease 4, with or without hepatic disease (263200)	AR	TOP
49	Genitourinary	*BRIP1*	c.2713_2715del (p.N905del) Exon5∼6del	Indel Deletion	Het Het	VUS LP	PM2_P + PM3+PM4 PVS1+PM2_P	Father Mother	Fanconi anemia, complementation group J (609054)	AR	Lost to follow-up
50	Genitourinary	*MYOCD*	c.934C>T (p. R312*)	Nonsense	Het	LP	PVS1+PM2	Father	Megabladder, congenital (618719)	AD	TOP
51	Genitourinary	*EYA1*	c.1090G>T (p.E364*)	Nonsense	Het	LP	PVS1+PM2_P	Mother	?Otofaciocervical syndrome (166780) Anterior segment anomalies with or without cataract (602588) Branchiootic syndrome 1 (602588) Branchiootorenal syndrome 1, with or without cataracts (113650)	AD	TOP
52	Genitourinary	*HNF1B*	c.336del (p.M113Cfs*12)	Frameshift	Het	P	PVS1+PS2_M + PM2_P	*De novo*	Renal cysts and diabetes syndrome (137920) Type 2 diabetes mellitus (125853)	AD	TOP
53	Genitourinary	*GREB1L*	c.554G>T (p.G185V)	Missense	Het	LP	PS2+PM2	*De novo*	Deafness, autosomal dominant 80 (619274) Renal hypodysplasia/aplasia 3 (617805)	AD	TOP
54	Hydrops + AFC-P	*PTPN11*	c.417G>C (p.E1390D)	Missense	Het	P	PS2_VS + PS3+PS4+PM2_P + PP2+PP3	*De novo*	LEOPARD syndrome 1 (151100) Noonan syndrome 1 (163950)	AD	TOP
55	Hydrops + Cardiovascular	*PTPN11*	c.1505C>T (p.S502L)	Missense	Het	P	PS2+PS4+PM2+PM5+PP2+PP3_M	*De novo*	LEOPARD syndrome 1 (151100) Noonan syndrome 1 (163950)	AD	Lost to follow-up
56	IncrNT/CH	*PTPN11*	c.922A>G (p.N308D)	Missense	Het	P	PS2+PS4+PM2_P + PP1_S + PP2+PP3_M	*De novo*	LEOPARD syndrome 1 (151100) Noonan syndrome 1 (163950)	AD	Lost to follow-up
57	IncrNT/CH	*KAT6B*	c.1146del (p.T383Hfs*74)	Frameshift	Het	LP	PVS1+PM2_P	Mother	Genitopatellar syndrome (606170) SBBYSS syndrome (603736)	AD	Live birth without any abnormality
58	IncrNT/CH + Cardiovascular	*RIT1*	c.270G>A (p.M90I)	Missense	Het	P	PS1+PS2+PS4_M + PM2+PP3	*De novo*	Noonan syndrome 8 (615355)	AD	TOP
59	Neurological	*L1CAM*	c.1003T>C (p.W335R)	Missense	Hemi	LP	PVS1+PM1+PM2+PP1-Strong + PP3	Mother	?Corpus callosum, partial agenesis of (304100) Hydrocephalus, congenital, X-linked (307000) MASA syndrome (303350)	XLR	TOP
60	Neurological	*B3GALNT2*	c.261–2A>G c.1453_1454delTG (p.W485Efs*8)	Splice acceptor Frameshift	Het Het	LP VUS	PVS1+PM2 PM2+PM4+PP3	Father Mother	Muscular dystrophy-dystroglycanopathy (congenital with brain and eye anomalies), type A, 11 (615181)	AR	TOP
61	Neurological	*L1CAM*	c.992-1G>A	Splice acceptor	Hemi	LP	PVS1+PM2_PP	Mother	?Corpus callosum, partial agenesis of (304100) Hydrocephalus, congenital, X-linked (307000) MASA syndrome (303350)	XLR	TOP
62	Neurological	*COL1A1*	c.4321G>T (p.D1441Y)	Missense	Het	P	PS2+PM2+PM1+PS3-pp + PP4	*De novo*	{Bone mineral density variation QTL, osteoporosis}(166710) Caffey disease (114000) Combined osteogenesis imperfecta and Ehlers-Danlos syndrome 1 (619115) Ehlers-Danlos syndrome, arthrochalasia type, 1 (130060) Osteogenesis imperfecta, type I (166200) Osteogenesis imperfecta, type II(166210) Osteogenesis imperfecta, type III(259420) Osteogenesis imperfecta, type IV(166220)	AD	TOP
63	Neurological	*PTEN*	c.518G>A (p.R173H)	Missense	Het	P	PS2+PS4_M + PM2_P + PP1+PP2+PP3	*De novo*	Cowden syndrome 1 (158350)	AD	Lost to follow-up
64	Neurological	*PTEN*	c.277C>G (p.H93D)	Missense	Het	P	PS4_P + PM1+PM2_P + PM5+PP3	*De novo*	Cowden syndrome 1 (158350)	AD	TOP
65	Neurological	*CHD7*	c.6034G>T (p.E2012*)	Nonsense	Het	P	PVS1+PS2_M + PM2_P	*De novo*	CHARGE syndrome (214800) Hypogonadotropic hypogonadism 5 with or without anosmia (612370)	AD	Lost to follow-up
66	Neurological	*DYNC1H1*	c.5885G>A (p. R1962H)	Missense	Het	P	PS2+PM2+PM1+PP3+pp5	*De novo*	Charcot-Marie-Tooth disease, axonal, type 2O (614228) Cortical dysplasia, complex, with other brain malformations 13 (614563) Spinal muscular atrophy, lower extremity-predominant 1, AD (158600)	AD	TOP
67	Neurological	*COL4A1*	c.3629G>C (p.G1210A)	Missense	Het	P	PS2_M + PM1+PM2+PP3	*De novo*	?Retinal arteries, tortuosity of (180000) Angiopathy, hereditary, with nephropathy, aneurysms, and muscle cramps (611773) Brain small vessel disease with or without ocular anomalies (175780) Microangiopathy and leukoencephalopathy, pontine, autosomal dominant (618564)	AD	Lost to follow-up
68	Neurological + AFV-P	*PNKP*	c.976G>A (p.E326K) c.1188 + 1G>A	Missense Splice donor	Het Het	P P	PS3+PM2-P + PM3+PP1_S PVS1+PM2-P + PM3	Father Mother	?Charcot-Marie-Tooth disease, type 2B2 (605589) Ataxia-oculomotor apraxia 4 (616267) Microcephaly, seizures, and developmental delay (613402)	AR	TOP
69	Neurological + Skeletal + Cardiovascular + USM-SUA	*FANCB*	c.1162del (p.Y388Tfs*7)	Frameshift	Hemi	LP	PVS1+PM2	Mother	Fanconi anemia, complementation group B (300514)	XLR	TOP
70	Situs inversus	*DNAH5*	c.6065T>C (p.L2022P) Exon4del	Missense Deletion	Het Het	VUS LP	PM2_P + PM3 PVS1+PM2_P	Mother Father	Ciliary dyskinesia, primary, 3, with or without situs inversus (608644)	AR	TOP
71	Skeletal	*COL1A1*	c.2164G>A (p.G722S)	Missense	Het	LP	PS2+PS4_P + PM1+PM2_P + PP3	*De novo*	{Bone mineral density variation QTL, osteoporosis}(166710) Caffey disease (114000) Combined osteogenesis imperfecta and Ehlers-Danlos syndrome 1 (619115) Ehlers-Danlos syndrome, arthrochalasia type, 1 (130060) Osteogenesis imperfecta, type I (166200) Osteogenesis imperfecta, type II(166210) Osteogenesis imperfecta, type III(259420) Osteogenesis imperfecta, type IV(166220)	AD	TOP
72	Skeletal	*ALPL*	c.1324C>T (p.Q442*) c.1334C>G (p.S445C)	Nonsense Missense	Het Het	LP VUS	PVS1-Strong + PM2 PM2+PM3+PP3	Mother Father	Hypophosphatasia, childhood (241510) Hypophosphatasia, infantile (241500)	AR	TOP
73	Skeletal	*EBP*	c.303G>A (p.W101*)	Nonsense	Het	P	PVS1+PS2_M + PM2_P	*De novo*	Chondrodysplasia punctata, X-linked dominant (302960) MEND syndrome (300960)	X-Link	Lost to follow-up
74	Skeletal	*FGFR3*	c.742C>T (p.R248C)	Missense	Het	P	PS2_VS + PS3_P + PS4+PM2_P + PP4	*De novo*	Achondroplasia (OMIM 100800) Hypochondroplasia (OMIM 146000)	AD	Lost to follow-up
75	Skeletal	*MYH3*	c.735T>G (p.F245L)	Missense	Het	LP	PM1+PM2_P + PP1+PP3+PP4	Father	Arthrogryposis, distal, type 2A (Freeman-Sheldon) (193700) Arthrogryposis, distal, type 2B3 (Sheldon-Hall) (618436) Contractures, pterygia, and spondylocarpostarsal fusion syndrome 1A (178110)	AD	TOP
76	Skeletal	*FGFR3*	c.1620C>A (p.N540K)	Missense	Het	P	PM2-PP + PS4+PS1	*De novo*	Achondroplasia (OMIM 100800) Hypochondroplasia (OMIM 146000)	AD	Lost to follow-up
77	Skeletal	*FGFR3*	c.746C>G (p.S249C)	Missense	Het	P	PS2+PS4+PM2_P + PP3_M	*De novo*	Achondroplasia (OMIM 100800) Hypochondroplasia (OMIM 146000)	AD	TOP
78	Skeletal	*FGFR3*	c.1138G>A (p.G380R)	Missense	Het	P	PS2_VS + PS4+PM2_P	*De novo*	Achondroplasia (OMIM 100800) Hypochondroplasia (OMIM 146000)	AD	TOP
79	Skeletal	*COL2A1*	c.1510G>A (p.G504S)	Missense	Het	P	PS2+PS4_M + PM1+PM2_P + PP1_S + PP3	*De novo*	?Epiphyseal dysplasia, multiple, with myopia and deafness (132450) ?Vitreoretinopathy with phalangeal epiphyseal dysplasia (619248) Achondrogenesis, type II or hypochondrogenesis (200610) Avascular necrosis of the femoral head (608805) Czech dysplasia (609162) Kniest dysplasia (156550) Legg-Calve-Perthes disease (150600) Osteoarthritis with mild chondrodysplasia (604864) Platyspondylic skeletal dysplasia, Torrance type (151210) SED congenita (183900) SMED Strudwick type (184250) Spondyloepiphyseal dysplasia, Stanescu type (616583) Spondyloperipheral dysplasia (271700) Stickler syndrome, type I (108300) Stickler syndrome, type I, nonsyndromic ocular (609508)	AD	TOP
80	Skeletal	*P3H1*	c.652G>T (p.E218*) c.454G>C (p.A152P)	Nonsense Missense	Het Het	P VUS	PVS1+PM2_P + PM3_P PM2 P + PM3	Father Mother	Osteogenesis imperfecta, type VIII(610915)	AR	Lost to follow-up
81	Skeletal	*COL3A1*	c.582 + 1G>A	Splice donor	Het	LP	PVS1+PM2_P	Mother	Ehlers-Danlos syndrome, vascular type (OMIM 130050)	AD	Lost to follow-up
82	Skeletal	*FGFR3*	c.1138G>A (p.G380R)	Missense	Het	P	PS2+PS4+PM2_P	*De novo*	Achondroplasia (OMIM 100800) Hypochondroplasia (OMIM 146000)	AD	TOP
83	Skeletal	*COL1A1*	c.2551G>A (p.G851S)	Missense	Het	LP	PS2 M + PM1+PM2 P + PP3	Father	{Bone mineral density variation QTL, osteoporosis}(166710) Caffey disease (114000) Combined osteogenesis imperfecta and Ehlers-Danlos syndrome 1 (619115) Ehlers-Danlos syndrome, arthrochalasia type, 1 (130060) Osteogenesis imperfecta, type I (166200) Osteogenesis imperfecta, type II(166210) Osteogenesis imperfecta, type III(259420) Osteogenesis imperfecta, type IV(166220)	AD	TOP
84	Skeletal	*DYNC2H1*	c.3842A>C (p.Y1281S) c.8833-1G>A	Missense Splice acceptor	Het Het	VUS LP	PM2_P PS3+PVS1_S + PM2_Supporting	Father Mother	Short-rib thoracic dysplasia 3 with or without polydactyly (OMIM 613091)	AR	TOP
85	Skeletal	*COL1A1*	c.1426G>A (p.G476R)	Missense	Het	P	PS2+PS4_P + PM1+PM2_P + PP3_M	*De novo*	{Bone mineral density variation QTL, osteoporosis}(166710) Caffey disease (114000) Combined osteogenesis imperfecta and Ehlers-Danlos syndrome 1 (619115) Ehlers-Danlos syndrome, arthrochalasia type, 1 (130060) Osteogenesis imperfecta, type I (166200) Osteogenesis imperfecta, type II(166210) Osteogenesis imperfecta, type III(259420) Osteogenesis imperfecta, type IV(166220)	AD	Lost to follow-up
86	Skeletal	*FANCA*	c.3989T>C (p.L1330P) Exon1∼6del	Missense Deletion	Het Het	VUS LP	PM2_P + PM3+PP3_M PVS1+PM2_P	Mother Father	Fanconi anemia, complementation group A (OMIM 227650)	AR	TOP
87	Skeletal	*ALPL*	c.98C>T (p.A33V) c.407G>A (p.R136H)	Missense Missense	Het Het	P P	PS3+PM2_P + PM3_S PS3+PM2_P + PM3_VS	Father Mother	Hypophosphatasia, adult (146300) Hypophosphatasia, childhood (241510) Hypophosphatasia, infantile (241500)	AR	Lost to follow-up
88	Skeletal	*FGFR3*	c.1620C>A (p.N540K)	Missense	Het	P	PS2+PS4+PM2_P	*De novo*	Achondroplasia (OMIM 100800) Hypochondroplasia (OMIM 146000)	AD	TOP
89	Skeletal	*FGFR3*	c.1138G>A (p.G380R)	Missense	Het	P	PS2+PS4+PM2_P	*De novo*	Achondroplasia (OMIM 100800) Hypochondroplasia (OMIM 146000)	AD	Lost to follow-up
90	Skeletal	*FGFR3*	c.742C>T (p.R248C)	Missense	Het	P	PS2 VS + PS3 P + PS4+PM1+PM2 P + PP3+PP4	*De novo*	Achondroplasia (OMIM 100800) Hypochondroplasia (OMIM 146000)	AD	TOP
91	Skeletal	*TNNT3*	c.188G>A (p.R63H)	Missense	Het	P	PS2+PS4+PM2_P + PP3_M + PP1_S	Mother	Arthrogryposis, distal, type 2B2 (618435)	AD	TOP
92	Skeletal	*PEX7*	c.337G>A (p.E113K) c.121G>C (p.G41R)	Missense Missense	Het Het	LP LP	PM2_P + PM3+PP1_M + PP3_M + PP4 PM2_P + PM3+PP1_M + PP3_M + PP4	Mother Father	Rhizomelic chondrodysplasia punctata, type 1 (215100)	AR	Lost to follow-up
93	Skeletal	*FGFR3*	c.1138G>A (p.G380R)	Missense	Het	P	PS2+PS4+PM2_P	*De novo*	Achondroplasia (OMIM 100800) Hypochondroplasia (OMIM 146000)	AD	TOP
94	Skeletal	*COL1A1*	c.1174G>C (p.G392R)	Missense	Het	LP	PS2+PM1+PM2_P + PP2+PP3	*De novo*	{Bone mineral density variation QTL, osteoporosis}(166710) Caffey disease (114000) Combined osteogenesis imperfecta and Ehlers-Danlos syndrome 1 (619115) Ehlers-Danlos syndrome, arthrochalasia type, 1 (130060) Osteogenesis imperfecta, type I (166200) Osteogenesis imperfecta, type II(166210) Osteogenesis imperfecta, type III(259420) Osteogenesis imperfecta, type IV(166220)	AD	TOP
95	Skeletal	*SETD5*	c.889_890del (p.L297Vfs*5)	Frameshift	Het	P	PVS1+PS2_M + PM2_P	*De novo*	Intellectual developmental disorder, autosomal dominant 23 (615761)	AD	TOP
96	Skeletal + AFV-P	*DYNC2H1*	c.7053_7054delTG (p.Cys2351Ter) c.8617A>G (p.Met2873Val)	Nonsense Missense	Het Het	P LP	PVS1+PM2+PP4 PM2+PM3+PP4+PP5	Mother Father	Short-rib thoracic dysplasia 3 with or without polydactyly (OMIM 613091)	AR	TOP
97	Skeletal + Cardiovascular	*EVC2*	c.2653C>T (nR885*) c1655_1658del (n.G552Dfs*2)	Nonsense Frameshift	Het Het	P P	PVS1+PM2 P + PM3 PVS1+PM2 P + PM3	Mother Father	Weyers acrofacial dysostosis (193530)	AR	TOP
98	Skeletal + Craniofacial	*FLNB*	c.1081G>T (p.G361C)	Missense	Het	P	PS2+PS4_P + PM2_P + PP3	*De novo*	Atelosteogenesis, type I (108720) Larsen syndrome (150250)	AD	TOP
99	Skeletal + Craniofacial	*RBM8A*	c.*6C>G (chr1:145413244_145826989)x1	3_prime_UTR Deletion	Het Het	LP P	Obtain Variant classification based on the genetic characteristics of the disease	Mother Father	Thrombocytopenia-absent radius syndrome (274000)	AR	TOP
100	Skeletal + Neurological	*FGFR3*	C.1138G>A (p.G380R)	Missense	Het	P	PS2+PS4+PM2_P	*De novo*	Achondroplasia (OMIM 100800) Hypochondroplasia (OMIM 146000)	AD	TOP
101	Skeletal + USM-ANB	*RUNX2*	c.1022–3090_*2442delinsC	Frameshift	Het	P	PVS1+PS2_M + PM2_P	*De novo*	Cleidocranial dysplasia (119600) Metaphyseal dysplasia with maxillary hypoplasia with or without brachydactyly (156510)	AD	TOP
102	USM2-UTD/VM	*FGFR3*	c.1138G>A (p.G380R)	Missense	Het	P	PS2+PS4+PM2_P	*De novo*	Achondroplasia (OMIM 100800) Hypochondroplasia (OMIM 146000)	AD	TOP
103	FGR	*GNAS*	c.486C>A (p.C162*)	Nonsense	Het	LP	PVS1+PM2_P	Father	Pseudopseudohypoparathyroidism (612463) Osseous Heteroplasia, progressive (166350)	AD	Lost to follow-up

Abbreviations: Hemi (hemizygosity); Het (heterozygosity); AD (autosomal dominant); AR (autosomal recessive); XLD (X-linked dominant); XLR (X-linked recessive); P (pathogenic); LP (likely pathogenic); TOP (Termination of Pregnancy).

Of the 454 enrolled fetuses, 152 did not undergo CMA. To estimate the overall additional diagnostic benefit of trio-WES compared to CMA, we calculated the diagnostic yield of CMA for the entire cohort. This estimate relied on the performance characteristics of the CMA platform used in our clinical laboratory, assuming conservatively that CMA would have detected all aneuploidies and pathogenic CNVs larger than 100 kb, which was the standard reporting threshold in our pipeline. It is important to note that this estimate may vary depending on the resolution. Among the 103 WES-positive cases, 26 were identified by CMA/karyotyping and trio-WES (Table 1), of which 25 were due to aneuploidies or CNVs larger than 100 kb that our CMA platform could detect (24 cases with CNVs or aneuploidies and 1 case of UPD), while the other case was missed by CMA; the remaining 77 diagnoses were identified solely through trio-WES (Table 2). Of these, 73 involved heterozygosities or compound heterozygosities with SNV/indel variations, and 4 involved compound heterozygosities with both SNV/indel and CNV loss variants. Of the four CNV-loss variants, three were smaller than 100 kb, and only one, measuring 413 kb, was detectable by CMA. In summary, the estimated yield of CMA was 5.7% (26/454), while that of trio-WES was 22.7% (103/454). Therefore, trio-WES provided an additional diagnostic yield of 17% over CMA.

### Molecular characteristics of cases diagnosed by trio-WES

The diagnostic sequence variants identified by WES were detailed in Table 2. The interpretation of the variants integrated clinical phenotypes and inheritance models to facilitate the assessment of fetal ultrasound results. These variants were interpreted based on the guidelines of the American College of Medical Genetics and Genomics (ACMG) and classified as pathogenic (P) or likely pathogenic (LP). Notably, 10 variants of uncertain significance (VUS), which were clinically relevant, were detected *in trans* with a P/LP variant in 10 cases associated with autosomal recessive disorders. Herein, all P/LP variants, as well as clinically relevant VUS, summarized in Table 2, were referred to as causal variants.

Of the 77 diagnosed cases, 52 (67.5%) were attributed to autosomal dominant (AD) disorders, 18 (23.4%) to autosomal recessive (AR) disorders, and 7 (9.1%) to X-linked disorders. Additionally, 73 cases were caused by SNV/INDEL variants, while 4 were compound heterozygotes of both SNV/INDEL and CNV variants. Among the 52 AD cases, 41 (78.8%) resulted from *de novo* variants. Eleven cases had inherited variants from each parent, four of whom (two paternal and two maternal) exhibited the indicated phenotype. Meanwhile, the parents of the remaining seven cases (four paternal and three maternal) without evident abnormal phenotypes suggested clinical heterogeneity or incomplete penetrance of the genetic variants. Of the 18 AR cases, only one (5.6%) was linked to a *de novo* variant along with a maternal heterozygous variant; the other 17 cases were compound heterozygotes. Among the seven X-linked cases, two (28.6%) were attributed to *de novo* variants, while the other five inherited cases included four instances (57.1%) of maternal inheritance and one instance (14.3%) of paternal mosaicism. (Table 2; Figure 3B).

### Gene-specific findings

Variants were identified in 47 distinct genes (Supplementary Figure S1). The most frequently implicated gene was *FGFR3*, which harbored pathogenic variants in 12 cases. These included the well-characterized missense variations c.1138G>A (p.G380R) in 6 cases, c.742C>T (p.R248C), and c.1620C>A (p.N540K) in 2 cases each, as well as single occurrences of c.746C>G (p.S249C) and c.749C>G (p.P250R). Notably, all *FGFR3* variants were confirmed as *de novo* variations by Sanger sequencing of parental blood samples. In addition to *FGFR3*, several other genes were involved in more than three cases, including *COL1A1* (n = 5), PTPN11 (n = 4), DYNC2H1 (n = 3), and COL2A1 (n = 3). Other genes were identified in two cases, such as *ALPL*, *CHD7*, *KMT2D*, *L1CAM*, *OCRL*, *PTEN*, *TSC1*, and *TSC2*. Further variants were found in single cases, as detailed in Supplementary Figure S1.

### Incidental findings

Variants unrelated to the primary indications for fetal testing, but potentially associated with severe childhood-onset diseases, were classified as incidental findings. A total of five cases were identified: three cases with AD variants (one maternal and two *de novo*), one with AR compound heterozygous variants, and one with an X-linked *de novo* heterozygous variant (Table 3).

**TABLE 3:** Incidental findings.

Case Id	Ultrasound findings	Gene	Variants	Molecular consequence	Zygosity	ACMG classification	ACMG criteria	Origin	OMIM diseases	Inheritancemode	Pregnancy outcome
104	Cardiovascular	*COL11A1*	c.1630-2del	Splice acceptor	Het	P	PVS1+PM2_P + PS4_P	Mother	Stickler syndrome, type II(604841)	AD	Lost to follow-up
105	Cardiovascular	*RASA1*	c.2365C>T (p.R789*)	Nonsense	Het	P	PVS1+PS4_M + PM2_P	*De novo*	Capillary malformation-arteriovenous malformation 1 (608354)	AD	TOP
106	Craniofacial	*PRRT2*	c.649dup (p.R217Pfs*8)	Frameshift	Het	P	PVS1+PS2_P + PS3_P + PP1_S + PP4	*De novo*	Convulsions, familial infantile, with paroxysmal choreoathetosis (602066) Episodic kinesigenic dyskinesia 1 (128200) Seizures, benign familial infantile, 2 (605751)	AD	Lost to follow-up
107	Genitourinary + USM-CPC	*ETFDH*	c.242T>C (p.L81P) c.1691–3C>G	Missense Splice_region	Het Het	LP LP	PM2+PM3+PP3+PP4 PM2+PM3+PP3+PP4	Mother Father	Glutaric acidemia IIC(231680)	AR	TOP
108	Situs inversus + Digestive + Cardiovascular	*ABCD1*	c.1415_1416del (p.Q472Rfs*83)	Frameshift	Het	P	PVS1+PS4_M + PM2_P	*De novo*	Adrenoleukodystrophy (OMIM 300100)	XLR	TOP

Abbreviations: Het (heterozygosity); AD (autosomal dominant); AR (autosomal recessive); XLR (X-linked recessive); P (pathogenic); LP (likely pathogenic); TOP (Termination of Pregnancy).

### Pregnancy outcomes

Pregnancy outcome data were available for 75 of the 103 cases diagnosed through CMA and trio-WES. Among these, 96.0% (72/75) of the pregnancies resulted in elective termination. It is important to note that these decisions were multifactorial, based on a comprehensive assessment that integrated the severity of the fetal ultrasound anomalies with the prognostic implications of the definitive genetic diagnosis. Two cases resulted in live births without detectable abnormalities, and one case resulted in a live birth with hydronephrosis (Tables 1,2). Long-term postnatal follow-up data to confirm genotype-phenotype correlations were not systematically available in this retrospective cohort, which represents a limitation for fully understanding the clinical spectrum of the diagnosed conditions. Four cases with incidental findings were selectively terminated (Table 3).

## Discussion

### Systematic analysis of diagnostic yields

This study evaluated the utility of trio-WES in prenatal scenarios, particularly for fetuses with ultrasound abnormalities. WES demonstrated an overall diagnostic yield of 22.7% for detecting genetic abnormalities associated with ultrasound anomalies, indicating a 17.2% increase in diagnostic yield compared to CMA. These findings emphasize the viability of WES as a critical diagnostic approach in prenatal diagnosis.

The diagnostic yield of WES in this study (22.7%) was consistent with prior reports, which range from 15% to 40% in cohorts of fetuses with structural anomalies identified by WES (Wapner et al., 2012; Robson et al., 2017; Levy and Wapner, 2018; Petrovski et al., 2019; Talkowski and Rehm, 2019; Fu et al., 2022; Qin et al., 2023; Margiotti et al., 2024). Notably, the highest diagnostic yield was observed in cases with skeletal system abnormalities (39.2%), aligning with prior reports of 30.4% (Fu et al., 2022), 39.1% (Wang et al., 2023), and 40% (Margiotti et al., 2024). These results surpass those reported in the PAGE study (15.4%) (Lord et al., 2019) and by Petrovski et al. (23.5%) (Petrovski et al., 2019). Multiple studies have reported diagnostic rates exceeding 50%, such as 56% (Qin et al., 2023) and 63.3% (Xiang et al., 2024). These variabilities are likely due to differences in phenotypic heterogeneity, inclusion criteria, sample sizes, and the classifications of genetic variants. The findings of this study also reinforced the observation that fetuses with multisystem anomalies exhibit higher diagnostic yields with WES (29.1%) compared to those with isolated anomalies, with this subgroup demonstrating the second-highest diagnostic rate, consistent with prior studies reporting yields ranging from 15.4% to 38.3% (Lord et al., 2019; Lai et al., 2022; Qin et al., 2023). This varied yield may be due to the broader and more complex genetic underpinnings involved, further supporting the effectiveness of WES in diagnosing complex congenital conditions.

The diagnostic yield and clinical utility of trio-WES varied significantly among prenatal cases with different phenotypes, highlighting the need for phenotype-driven diagnostic approaches. For fetuses with abnormal amniotic fluid volume, CMA alone achieved diagnostic rates of 14.3%, and trio-WES did not significantly improve the diagnostic yield, indicating that CMA remains the preferred initial test. Conversely, application of trio-WES further increased the identification of phenotype-related causal variants in subgroups such as skeletal system anomalies (increased by 40%), multisystem anomalies (increased by 12.5%), genitourinary system anomalies (increased by 5.9%), and IncrNT/CH (increased by 5.6%), emphasizing the importance of applying both CMA and trio-WES together in relevant prenatal cases. Moreover, trio-WES provided the sole diagnosis in several phenotypic subgroups where CMA was non-diagnostic for identifying phenotype-related causal variants in fetuses with craniofacial anomalies, neurologic anomalies, cardiovascular anomalies, and situs inversus, thereby advocating for the incorporation of trio-WES in these phenotypes. Although these observations were based on limited sample sizes and require validation, they suggested that trio-WES can uncover the genetic etiology in a subset of these cases that would otherwise remain undiagnosed. Nonetheless, WES failed to detect any pathogenic variants in fetuses with gastrointestinal, abdominal, or respiratory system anomalies, consistent with previously reported low diagnostic yields in cases with the same phenotypes (Lord et al., 2019; Qin et al., 2023; Wang et al., 2024), suggesting a limited utility of WES in such cases.

In the hydrops fetalis subgroup, WES did not identify any clinically relevant variants. This finding contrasts with previous studies reporting diagnostic yields of 9.1% (Lord et al., 2019) and 47.8% (Xiang et al., 2024). This discrepancy may be related to differences in case classification and inclusion criteria. In the present study, hydrops fetalis was defined strictly as the presence of two or more abnormal fluid collections (e.g., ascites, pericardial effusion, pleural effusion, or skin edema) detected via prenatal ultrasound. Cases featuring isolated increased NT or accompanied by a single abnormal fluid collection were categorized under the increased NT/cystic hygroma subgroup.

Additionally, while a previous study utilizing WES in 246 cases of unexplained stillbirth reported a diagnostic yield of 8.5% (Stanley et al., 2020), the present study did not identify clinically relevant variants in eight stillbirth samples. Given the highly heterogeneous etiology of stillbirth, which includes genetic and non-genetic factors such as maternal health conditions, placental abnormalities, and environmental influences (Silver and Reddy, 2024), this implied that a larger sample size might be required to address the diagnostic differences.

### Application of Trio-WES in multiple specific clinical scenarios

Beyond evaluating trio-WES in the context of broad structural anomalies, we explored its utility in fetuses with cardiac rhabdomyoma and isolated crystalline anomalies. These phenotypes exhibited higher diagnostic rates compared to other subgroups. Although the sample size was limited, the results suggest that trio-WES holds significant potential for providing diagnostic insights into these specific isolated anomalies. The ongoing development of prenatal imaging and the identification of links between prenatal phenotypes and genotypes of genetic diseases indicate that trio-WES is poised to enhance the diagnostic yield of genetic variations in fetuses presenting with structural abnormalities prenatally. Furthermore, the application of trio-WES could be extended to various other specific phenotypes, including severe hydrocephalus, periventricular heterotopia, and thanatophoric dysplasia, among others.

*FGFR3* and *COL1A1* were the most commonly associated genes with skeletal anomalies, aligning with the high diagnostic rates observed for these conditions. Notably, all variations identified in the *FGFR3* gene were confirmed as *de novo* variations in this study, presenting distinct challenges in clinical genetics. However, fetuses harboring *de novo* variations in the *FGFR3* gene did not exhibit a propensity for advancement due to paternal age (data not shown). In this study, *de novo* variants accounted for 78.8% (41/52) of causal variants in AD disease genes and 28.6% (2/7) of causal variants in X-linked disease genes, which was consistent with previous research findings reporting *de novo* variations of 64%∼87.1% in AD genes and 11%∼12.9% in X-linked genes (Posey et al., 2017; Jarvela et al., 2021). The occurrence of *de novo* variants linked to autosomal dominant genetic disorders is stochastic and unpredictable, posing challenges to prenatal diagnosis. Certain variants did not manifest discernible phenotypes prenatally, presenting a significant obstacle to early disease detection. Moving forward, noninvasive prenatal screening for monogenetic disorders holds considerable clinical promise for recognizing a broader spectrum of *de novo* variants linked to autosomal dominant conditions at their incipient stages (Brand et al., 2023).

In a fetus with polyhydramnios, CMA identified two regions of homozygosity (ROH) on chromosome 15q, excluding any imprinted genes. Subsequent trio-WES elucidated the origin of the ROH, confirming a mixed condition of maternal UPD15 in this case, with isodisomy in the ROH segments and heterodisomy in the remainder of chromosome 15, leading to the manifestation of Prader-Willi syndrome. While further use of trio-WES helped prevent oversights regarding the fetal anomaly, these results underscore the pivotal role of trio-WES in scenarios involving ROH identified by CMA.

Five cases in our cohort revealed incidental genetic findings. Three involved autosomal dominant (AD) variants: Case 108 had a variant linked to Type II Stickler syndrome (MIM: 604841); Case 109 had a variant associated with capillary malformation-arteriovenous malformation 1 (MIM: 608354); and Case 110 carried a variant related to ICCA syndrome (MIM: 602066), episodic kinesigenic dyskinesia 1 (MIM: 128200), and benign familial infantile seizures 2 (MIM: 605751). Additionally, one case involved compound heterozygous variants associated with glutaric acidemia type IIC (MIM: 231680), and an X-linked variant was connected to adrenoleukodystrophy (MIM: 300100). Although these findings were unrelated to the presenting fetal phenotypes, they indicated potential for moderate to severe diseases, with onset ranging from the neonatal period through childhood and into adulthood. The detection of such incidental findings emphasizes the importance of comprehensive and systematic genetic counseling when performing exome sequencing. In accordance with ACMG recommendations and our institutional protocol, the possibility of incidental findings was thoroughly discussed during pre-test genetic counseling, and written informed consent was obtained. For the five cases identified, these findings were disclosed to the parents in a dedicated post-test counseling session. Since these findings were unrelated to the primary fetal phenotype, they did not directly impact pregnancy management decisions; the four terminations were primarily due to the severity of ultrasound anomalies and the associated primary genetic diagnoses. This process highlights the need for a strong ethical framework to ensure responsible use of prenatal exome sequencing.

### The essential role of Trio-WES in prenatal diagnosis: a phenotype-driven approach and its implications for genetic counseling and pregnancy outcomes

The findings from our large cohort of 454 fetuses enable us to propose a refined, phenotype-driven approach for the clinical use of trio-WES, moving from theoretical potential to data-supported application. Our results indicate that the diagnostic value of trio-WES depends on the specific ultrasound anomaly observed. This suggests shifting away from a one-size-fits-all method toward a detailed protocol that improves diagnostic accuracy and clinical outcomes.

First, our data provide clear evidence for a tiered diagnostic strategy. The exceptionally high diagnostic yields in fetuses with skeletal system anomalies and multisystem anomalies, along with the significant incremental yield over CMA, strongly support the simultaneous use of trio-WES with chromosomal analysis in these categories. Moreover, trio-WES demonstrated exclusive diagnostic ability for several phenotypic subgroups where CMA failed to identify clinically relevant variants, including craniofacial anomalies (12.5%), neurological anomalies (10%), cardiovascular anomalies (5.6%), and situs inversus (20%). Although the additional yield over CMA for neurological anomalies was not statistically significant, the fact that trio-WES provided the only diagnosis in many of these cases supports its incorporation into the diagnostic process for these phenotypes. For fetuses with these specific anomalies—especially when a monogenic disorder is strongly suspected based on ultrasound findings—our data advocate using trio-WES either sequentially or alongside CMA to maximize diagnostic efficiency and prevent delays. Conversely, for anomalies such as isolated amniotic fluid volume abnormalities, where trio-WES did not significantly increase the CMA yield, CMA remains the appropriate initial test. This phenotype-driven approach ensures efficient use of resources and maximizes diagnostic yield.

Second, our study emphasizes the importance of trio-WES in identifying specific causes. For cases with cardiac rhabdomyoma or isolated crystalline lens anomalies, which have very high diagnostic rates (57.1% and 60.0%, respectively), trio-WES is crucial for confirming monogenic disorders such as tuberous sclerosis or congenital crystalline lens anomalies. Additionally, when CMA detects regions of homozygosity or absence of heterozygosity (ROH/AOH), trio-WES offers an important follow-up step by determining the parental origin of the alleles. As shown in Case 24, this ability is vital for confirming or ruling out the diagnosis of UPD related to imprinted disorders, moving beyond initial ROH detection to a definitive molecular diagnosis. This method helps avoid diagnostic oversights and provides a more accurate assessment of the risks associated with ROH findings.

Finally, and most importantly, the molecular diagnoses provided by trio-WES significantly influenced clinical management and parental counseling. The clear result that 96.0% (72/75) of pregnancies with a positive diagnosis led to elective termination highlights the vital role a definitive genetic finding plays in parental decision-making for severe fetal conditions. The high proportion of *de novo* variants (78.8% in autosomal dominant disorders) provided families with straightforward, low recurrence risk information, which is often reassuring for future reproductive planning. In contrast, identifying inherited variants, including those from asymptomatic parents with incomplete penetrance or mosaicism, required more complex counseling about family implications and personalized recurrence risks. Managing incidental findings, although rare, further underscores the importance of thorough pre-test counseling to prepare families for all possible outcomes. Therefore, beyond its diagnostic capabilities, trio-WES is a crucial tool for delivering personalized genetic counseling, supporting informed reproductive choices, and guiding perinatal management.

In conclusion, our findings support integrating trio-WES into a comprehensive prenatal diagnostic framework, where its application is strategically guided by fetal phenotype. This approach notably enhances diagnostic accuracy and, in turn, provides reliable information essential for effective clinical management and compassionate, evidence-based genetic counseling.

## Limitations and future directions

Although our study provided strong evidence supporting the effectiveness of trio-WES in prenatal diagnostics, several limitations need to be addressed. First, the retrospective nature of the study hinders the establishment of causal relationships and a thorough evaluation of the long-term clinical implications of WES findings on pregnancy outcomes. Crucially, the absence of systematic longitudinal postnatal follow-up data limits our understanding of the full phenotypic spectrum and long-term prognosis associated with the genetic diagnoses made prenatally. Second, the interpretive challenges inherent to WES must be acknowledged. Managing VUS, especially *de novo* VUS, presents a significant counseling challenge, potentially causing parental anxiety and decision-making paralysis without clear prognostic information. Similarly, the identification of incidental findings, while managed through informed consent in this study, highlights ongoing ethical and counseling challenges in handling information unrelated to the initial diagnostic question. Third, our statistical comparison of WES and CMA did not adjust for potential confounding variables such as gestational age or phenotypic severity. While this approach provides a direct comparison of diagnostic yield, future studies with larger cohorts could employ multivariate analyses to control for such factors. Fourth, the relatively small sample sizes within specific phenotypic subgroups, such as situs inversus, cardiac rhabdomyoma, and crystalline lens anomalies, may limit the generalizability of our results. Our conclusions for these subgroups should therefore be interpreted as preliminary and descriptive. Future prospective studies with larger, more multicenter cohorts, combined with standardized postnatal follow-up protocols, are essential to validate these findings, clarify the clinical significance of prenatally identified variants, and enhance genotype-phenotype correlations. A significant drawback of trio-WES is its inability to reliably identify major balanced structural variations or variants in non-coding regions. To address these limitations and enhance diagnostic accuracy, combining WES with other methodologies such as whole-genome sequencing (WGS), long-read genome sequencing, or optical genome mapping (OGM) (Qu et al., 2023) could provide a more comprehensive understanding of the genetic framework underlying fetal structural anomalies.

## Conclusion

This study expanded the use of exome sequencing in prenatal diagnostics by including various phenotypic categories and developing diagnostic strategies for different prenatal situations. These results demonstrated the effectiveness of prenatal diagnosis, especially for fetuses with ultrasound abnormalities, and identified clinical applications and strategies for trio-WES use. Additionally, creating comprehensive genotype-phenotype databases is vital for improving the diagnostic ability of trio-WES, enhancing variant interpretation, and supporting personalized genetic counseling for prospective parents.

## Data Availability

The original contributions presented in the study are included in the article/Supplementary Material, further inquiries can be directed to the corresponding author.
