# Supplementary material for: Application of trio-based whole-exome sequencing in fetal ultrasound anomalies: a single-center retrospective study of 454 cases
Source: Front Genet. 2025 Nov 24;16:1662801. doi: 10.3389/fgene.2025.1662801 (PMC12682908; doi:10.3389/fgene.2025.1662801)
Supplement: Supplementary file 1 [file Table1.docx]

**Sumpplementary Table 1. The information of 9 FGFR3 mutations.**

| Case ID | Ultrasound findings | Gene | Variants | Zygosity | ACMG classification | ACMG criteria | Origin |
| --- | --- | --- | --- | --- | --- | --- | --- |
| 38 | Craniofacial | FGFR3 | c.749C>G(p.P250R) | het | P | PS2_VS+PS4+PM2_P+PP1_S+PP3 | De novo |
| 74 | Skeletal | FGFR3 | c.742C>T(p.R248C) | het | P | PS2_VS+PS3_P+PS4+PM2_P+PP4 | De novo |
| 76 | Skeletal | FGFR3 | c.1620C>A（p.N540K） | het | P | PM2-PP+PS4+PS1 | De novo |
| 77 | Skeletal | FGFR3 | c.746C>G（p.S249C） | het | P | PS2+PS4+PM2_P+PP3_M | De novo |
| 78 | Skeletal | FGFR3 | c.1138G>A(p.G380R) | het | P | PS2_VS+PS4+PM2_P | De novo |
| 82 | Skeletal | FGFR3 | c.1138G>A(p.G380R) | het | P | PS2+PS4+PM2_P | De novo |
| 88 | Skeletal | FGFR3 | c.1620C>A(p.N540K) | het | P | PS2+PS4+PM2_P | De novo |
| 89 | Skeletal | FGFR3 | c.1138G>A（p.G380R） | het | P | PS2+PS4+PM2_P | De novo |
| 90 | Skeletal | FGFR3 | c.742C>T(p.R248C) | het | P | PS2 VS+PS3 P+PS4+PM1+PM2 P+PP3+PP4 | De novo |
| 93 | Skeletal | FGFR3 | c.1138G>A（p.G380R） | het | P | PS2+PS4+PM2_P | De novo |
| 100 | Skeletal+Neurological | FGFR3 | C.1138G>A(p.G380R) | het | P | PS2+PS4+PM2_P | De novo |
| 102 | Skeletal+USM3-UTD/VM/EIF | FGFR3 | c.1138G>A(p.G380R) | het | P | PS2+PS4+PM2_P | De novo |
